# Clinical Benefits of Therapeutic Interventions Targeting Mitochondria in Parkinson’s Disease Patients

**DOI:** 10.2174/1871527322666230330122444

**Published:** 2024-02-01

**Authors:** Matteo Ciocca, Chiara Pizzamiglio

**Affiliations:** 1 Department of Brain Sciences, Imperial College London, London, UK;; 2 Department of Neuromuscular Diseases, UCL Queen Square Institute of Neurology, London, UK

**Keywords:** Parkinson’s disease, treatment, mitochondria, supplements, neuroprotection, oxidative stress, mitochondrial dysfunction

## Abstract

Parkinson’s disease is the second most common neurodegenerative disease. Mitochondrial dysfunction has been associated with neurodegeneration in Parkinson’s disease, and several treatments targeting mitochondria have been tested in these patients to delay disease progression and tackle disease symptoms. Herein, we review available data from randomised, double-blind clinical studies that have investigated the role of compounds targeting mitochondria in idiopathic Parkinson’s disease patients, with a view of providing patients and clinicians with a comprehensive and practical paper that can inform therapeutic interventions in this group of people. A total of 9 compounds have been tested in randomized clinical trials, but only exenatide has shown some promising neuroprotective and symptomatic effects. However, whether this evidence can be translated into daily clinical practice still needs to be confirmed. In conclusion, targeting mitochondrial dysfunction in Parkinson’s disease is a promising therapeutic approach, although only one compound has shown a positive effect on Parkinson’s disease progression and symptoms. New compounds have been investigated in animal models, and their efficacy needs to be confirmed in humans through robust, randomised, double-blind clinical trials.

## 1 INTRODUCTION

Parkinson’s disease (PD) is the most common neurodegenerative movement disorder, with an estimated prevalence in Europe of 108-257/100000 [1, 2]. The pathological hallmark of PD is the progressive loss of dopaminergic neurons alongside the deposition of alpha-synuclein [3]. The classic clinical symptoms (*i.e*., bradykinesia, rigidity, and rest tremor) become evident when more than 70% of the dopaminergic neurons of the substantia nigra pars compacta are lost [4].

Evidence supports oxidative stress's relevance in leading the dopaminergic neurodegeneration in all forms of PD [5, 6]. Importantly, oxidative stress has been described in patients with early-stage PD, suggesting a causative role rather than a secondary effect in dopaminergic neurodegeneration [6]. Mitochondrial dysfunction is one of the leading contributors to oxidative stress, which has been well elucidated in PD [6]. The cause of oxidative stress, and this is an imbalance between the production and clearance of oxygen reactive species (ROS), resulting in ROS accumulation. ROS overproduction leads to mitochondrial damage through several mechanisms, including mitochondrial DNA mutations, damage to the mitochondrial respiratory chain, alteration of membrane permeability, and Ca^2+^ homeostasis [7]. Abnormalities in mitochondrial function, turnover, dynamics, and metabolism contribute to neuronal malfunction, neurodegeneration, and protein aggregation and deposition [8]. Therefore, targeting mitochondrial dysfunction may be a promising therapeutic approach to tackle motor symptoms and delay disease progression by protecting and restoring neurodegeneration of dopaminergic neurons, especially in the early PD stage [9].

Several compounds aiming to improve mitochondrial dysfunction have been investigated in pre-clinical studies of PD, and most of them have shown promising neuroprotective and symptomatic effects [8, 10]. Some of these compounds are already available on the market as pharmacological treatments for other conditions but PD [11], whereas others are accessible as over-the-counter food supplements [12].

Approximately between 25.7% and 76% of patients with PD use at least one complementary treatment, mostly suggested by a family member or identified using media and social media [13, 14]. Medications designed for other conditions, herbal medicines, and vitamin supplements are the most used complementary treatments both in the Eastern than in Western countries [14].

In this mini-review, we focused on the current evidence about the clinical efficacy in sporadic PD of drugs/supplements targeting mitochondrial dysfunction. We limited our review to human studies, reporting data from randomised, double-blind clinical studies (RCTs). We hope that such a targeted review can expand the knowledge of patients and clinicians on the current evidence for treatments targeting mitochondrial dysfunction. A detailed description of mitochondrial dysfunction in PD is not in the scope of this paper and can be found elsewhere [15, 16].

## 2 METHODS

Initially, we identified a list of possible alternative pharmacological treatments and food supplements that may improve any mitochondrial abnormalities identified in PD. To achieve this, we conducted a thorough literature search initially using the following combinations of terms on the MEDLINE database: “mitochondrial dysfunction and Parkinson’s disease”, “Parkinson's disease and food supplements”, “mitochondrial dysfunction and Parkinson’s disease and food supplements” and “mitochondrial dysfunction and Parkinson’s disease and pharmacological treatment”. Our search was limited to articles published between 1982 and 2022. Subsequently, we cross-checked our findings with findings reported in some of the most recently published comprehensive reviews on pharmacological treatments or food supplements targeting mitochondrial dysfunction in PD [10, 17-21]. The list of compounds identified is available in Table **1**.

As some identified compounds may have been tested only on cellular and/or animal models, we ran a second literature research on the MEDLINE database. We filtered our results by using the species filter “HUMAN” available on MEDLINE, and we focused only on RCTs written in English and identified by using the terms listed in Table **1** followed by “Parkinson’s disease”, linked by the “AND” function. We decided to include only RCTs to ensure that possible suggestions to clinicians were based only on evidence from high-quality articles.

Finally, we searched the clinicaltrial.gov database to identify ongoing trials targeting mitochondrial dysfunction in PD using one of the compounds listed in Table **1**.

## 3 RESULTS

After the first search, we identified 18 possible compounds that impacted PD motor symptoms, exerted a neuroprotective effect, or possibly reduced mitochondrial dysfunction. After filtering for human studies and RCTs, we identified eleven compounds tested in RCTs. For nine of them, results have been published and discussed in this review.

Five of the remaining 7 compounds were excluded because they have been studied only in cellular or animal models or have not been tested in RCTs. Similarly, the other two compounds have been suggested as possibly effective in targeting mitochondrial dysfunction in PD. However, no studies on their effect on cellular or animal models are available. For each compound, a detailed breakout of the total number of studies identified, the number of closed and published RCTs on PD patients, and the ongoing clinical trial identified on the clinicaltrial.gov database are provided in Table **1**.

We divided the compounds according to their activity on the mitochondrial function: antioxidant effect, metabolic effects, mitochondrial biogenesis, and effects on calcium homeostasis. A summary of published RCTs is available in Table **2**.

### 3.1 Antioxidant Effects

#### 3.1.1 Coenzyme Q10

Coenzyme Q10, or ubiquinone, is a fat-soluble compound that transports electrons in the inner mitochondrial membrane, facilitating oxidative phosphorylation [22]. It also enhances complex I activity and has powerful antioxidant properties by protecting mitochondrial membranes from oxidative stress [23]. Several studies have investigated the effects of supplementation with a high, moderate, and low daily dose of Coenzyme Q10.

The first study on Coenzyme Q10 in early PD patients showed a disability reduction in patients receiving high-dose supplementation (up to 1200 mg daily), whereas no changes were observed in low and medium doses (300 and 600 mg daily, respectively) [24]. A mild reduction in the total Unified Parkinson Disease Rating Scale (UPDRS) score after 4 weeks of low-to-moderate dose supplementation (360 mg daily) was also reported later by another group [25]. However, no significant difference *versus* placebo was reported. In 2007, Storch and collaborators ran an RCT on 106 PD patients, 51 of them treated with a low daily dose (300 mg) of Coenzyme Q10 for 3 months. No significant improvement in the UPDRS-III scores was noted [26].

Following these results, the Parkinson Study Group QE3 ran an RCT of high-dosage coenzyme Q10 supplementation (1200 or 2400 mg daily) on 267 early PD patients, of which 173 were on treatment. No significant clinical improvement using the total UPDRS score was noted after 16 months [27]. Similar results on early onset drug-naïve PD patients were reported by Yoritaka and colleagues in 2015 on a cohort of 14 patients who took a reduced form of CoQ10 (ubiquinol-10, 300 mg) for 96 weeks [28]. In the same study, 14 PD patients experiencing wearing OFF reported improved motor symptoms [28].

Finally, Coenzyme Q10 has been associated with creatine in a trial to evaluate possible neuroprotective effects on cognitive functions in PD patients with mild cognitive impairment [29]. Seventy-five patients were randomly treated with a combination of creatine 10 g daily and Coenzyme Q10 300 mg daily or with a placebo. The combination therapy delayed the cognitive decline as measured using the Montreal cognitive assessment, suggesting a possible neuroprotective effect on non-motor symptoms in PD [29].

#### 3.1.2 Mitoquinone

Mitoquinone (MitoQ) is a synthetic mitochondria-targeted antioxidant, which PD patients tolerate well. In a double-blind, placebo-controlled study, MitoQ was administered for one year in two different dosages in a total of 89 patients, whereas 41 patients received a placebo [30]. The primary endpoint was the total MDS-UPDRS. Secondary endpoints included the Hoehn & Yahr score, Schwab and England Scale, timed hand-tapping score, the Mini-Mental State Examination, and the time to disability requiring anti-parkinsonian medication. No difference between MitoQ and placebo on any measure of PD progression was observed [30].

#### 3.1.3 N-acetyl Cysteine

N-acetyl cysteine (NAC) is available over the counter as an oral supplement, and it has antioxidant properties by increasing the level of blood glutathione. A pilot study has shown some promising effects on PD motor symptoms [31]. No RCTs have been published on NAC as a symptomatic or neuroprotective treatment in PD. However, a randomised, cross-over interventional trial is ongoing (NCT04459052).

#### 3.1.4 Inosine

Urate is an endogenous antioxidant that acts on mitochondrial biogenesis *via* the Nrf2 antioxidant response pathway. Urate is an inverse risk factor for PD in healthy subjects, and PRECEPT (Parkinson Research Experiment on CEP-1347 Trial) and DATATOP (Deprenyl and Tocopherol Antioxidative Therapy of Parkinsonism) trials showed that higher serum urate was associated with slower clinical progression of PD [32, 33].

In the Safety of URate Elevation in PD trial [34], the urate precursor inosine was administered to 75 PD patients, and a good safety and tolerability profile was shown. Subsequently, in a larger RCT to assess neuroprotective effects, 149 drug-naïve PD patients with low serum urate levels were treated with inosine to increase urate levels between 7.1-8.0 mg/dL. The study closed early as clinical progression was similar between participants randomised to inosine and placebo [35]. In conclusion, no evidence supports inosine as a neuroprotective treatment for PD.

#### 3.1.5 Nicotinamide Riboside

Nicotinamide adenine dinucleotide (NAD^+^) is central to mitochondrial homeostasis, and there is growing evidence of a deficit of NAD^+^ in PD [36]. Restoring levels of NAD^+^ *via* supplementation of nicotinamide riboside (NR) can potentially improve PD symptoms by restoring the mitochondrial respiratory dysfunction [36, 37]. In a phase I trial, Brakedal and colleagues described 31 patients with PD randomly assigned to 1 gr of NR or placebo [38]. Results showed a good safety profile for NR, leading to increased cerebral NAD levels (measured by [31] phospohorous-magnetic resonance spectroscopy) in 10 out of 13 patients assigned to the NR group.

Despite no differences in MDS-UPDRS being noticed between the two groups, a trend in a reduction for the MDS-UPDRS was observed in patients with >10% increase in cerebral NAD levels [38].

#### 3.1.6 Curcumin

Curcumin protects mitochondrial functions through several mechanisms, but mainly by restoring mitochondrial membrane potential [39]. Ghodsi and collaborators recently investigated the effect of curcumin on PD motor symptoms and quality of life in a randomised, triple-blind, placebo-controlled trial. No significant differences between treated and untreated groups were observed [40].

#### 3.1.7 Ursodeoxycholic Acid (UDCA)

In animal models, UDCA has been shown to protect dopaminergic neurons from oxidative stress by regulating mitochondrial dysfunction [41, 42]. In a pilot study on five individuals with PD, findings suggested that UDCA was generally well-tolerated, with modest improvements in ATP levels and a decrease in ATPase activity. ATPase activity, and a mild in the total UPDRS score, highlighting the need for further research [43]. A two-centre, RCT, placebo-controlled trial is currently ongoing [44]. In this phase II clinical trial, researchers aim to evaluate the safety and tolerability of 30 mg/kg of UDCA in 30 participants with early PD. The trial also aims to confirm the target engagement of UDCA using ^31^Phosphorus MR Spectroscopy to assess the levels of ATP and relevant metabolites in the brain. Effects on motor impairment will be measured with the UPDRS score and using wearable sensors in supervised and unsupervised real-life conditions [44]. Recruitment for this study is closed, but results are not available yet.

### 3.2 Metabolic Effects

#### 3.2.1 Creatine

Creatine plays an important role in mitochondrial energy production, and dietary supplements of creatine monohydrate have been used for many decades by young athletes to improve performance [45]. A total of 5 randomised controlled trials have been published.

In 2006, Bender and colleagues conducted a 2-year placebo-controlled randomised clinical trial in 60 PD patients [46]. A loading dose of 20 gr/daily for 6 weeks, followed by 6 months at 2 gr/daily and then at 4 gr/daily for the remaining period, was administered. No differences in motor symptoms and quality of life between the active and placebo group were noted [46]. Later, a larger RCT involving 1741 PD patients showed similar negative results on motor and cognitive symptoms, the activity of daily living, disability, and quality of life [12]. Interestingly, a potentially harmful interaction between caffeine and creatine was observed, as higher caffeine intake was associated with faster progression in patients taking creatine [47].

### 3.3 Effects on Mitochondrial Biogenesis

#### 3.3.1 Exenatide

Pre-clinical studies showed the neuroprotective effects of exenatide, a GLP-1 receptor agonist currently licensed for type 2 diabetes mellitus. However, exenatide passes the blood-brain barrier, and it has been shown to stimulate mitochondrial biogenesis and exert neuroprotection *via* GLP-1 receptors [48].

In a proof-of-concept, single-blind trial, exenatide was well-tolerated by PD patients, and an improvement in the MDS-UPDRS after 1 year of treatment was reported in the treated group [49]. This led to a single-centre, randomised, double-blind, placebo-controlled trial targeting patients on levodopa replacement therapy and with wearing-off effects [50]. Weekly subcutaneous injections of exenatide 2 mg showed an improvement in the total UPDRS-III score measured in the OFF state in the treated group after 48 consecutive weeks of treatment. Interestingly, the improvement persisted for 12 weeks after the end of the administration period, suggesting that exenatide may have both a neuroprotective and an acute effect on motor symptoms [50].

In addition, two post-hoc analyses showed that (1) exenatide led to improvements in mood and general well-being [51], and (2) tremor-dominant phenotype and lower MDS-UPDRS-II scores were the best predictors of motor improvement to exenatide, whereas older PD patients and patients with disease duration longer than 10 years were poor responders to the treatment [52]. To confirm the clinical improvement and explore whether there is a disease modifying effect, a larger multicentre RCT has been designed and is ongoing [53].

#### 3.3.2 Pioglitazone

Pioglitazone is another medication licensed to treat type 2 diabetes mellitus. The NET-PD network identified pioglitazone as one possible disease-modifying agent for PD [54]. Pioglitazone is a peroxisome proliferator-activated receptor γ (PPAR-γ) agonist, and it exerts control on mitochondrial biogenesis and antioxidant effect through interaction with its transcriptional coactivator PPAR-γ coactivator 1-α (PGC-1α). In a phase II RCT, the NET-PD group investigated pioglitazone in 210 early PD patients, randomly assigned to 15 mg/day, 45 mg/day of pioglitazone, or placebo. Findings did not support the hypothesis of a neuroprotective effect of pioglitazone at these specific doses [54].

### 3.4 Effects on Calcium Homeostasis

#### 3.4.1 Isradipine

Isradipine is a calcium channel blocker that is thought to have dose-dependent neuroprotective effects by acting on CAV-1 L-type calcium channels in the substantia nigra [11]. In an RCT phase 3 study involving 336 patients with early PD, isradipine failed to show any neuroprotective effect using changes in the MDS-UPDRS part I to III measured in ON state at baseline and after 36 months [55]. However, the secondary analysis of pharmacokinetic data showed that the need for initiating antiparkinsonian medication was delayed when isradipine exposure increased [56]. This was also confirmed by a re-analysis of the phase II clinical trial on isradipine [57], suggesting that a dose-dependent neuroprotective effect is possible in PD patients. However, additional studies are necessary to confirm this hypothesis.

## 4 DISCUSSION

Targeting mitochondrial dysfunction in PD has been considered a promising field, mainly because of the contribution of mitochondria to oxidative stress. However, despite being generally safe and well tolerated, the effectiveness of these compounds on idiopathic PD has been inconsistent, and results are often disappointing. This may be due to the heterogeneity of PD, the duration of clinical trials, and/or the choice of appropriate biomarkers. In this paper, we discussed the potential effects of 11 compounds in targeting mitochondrial dysfunction in PD patients. Nine compounds have been tested in RCTs, whereas 2 compounds have shown promising preliminary results in pilot studies, and RCTs are ongoing. Seven compounds showed no significant neuroprotective or symptomatic effect on motor symptoms in PD patients. Coenzyme Q10 has shown some questionable results within these compounds, but no convincing evidence of neuroprotection emerges after considering all the published data. Conversely, exenatide seems to be the most promising treatment, as it showed improvement in the UPDRS-III in two different RCTs and patients with moderate PD severity. An ongoing trial will investigate whether any neuroprotective effect is present in early PD patients. Exenatide is a GLP-1 receptor agonist. GLP-1 receptors have been identified in different brain areas, and *in vitro* and *in vivo* studies have suggested that exenatide may have neuroprotective and neurorestorative effects [49]. The exact mechanism of these effects is uncertain, but it may be related to neurotrophic effects, anti-inflammatory properties, facilitation of neurogenesis, or stimulation of mitochondrial biogenesis [49]. Studies in animal models have shown the ability of exenatide to cross the blood-brain barrier at dosages similar to those used in type 2 diabetes [50]. In addition, animal studies showed that exenatide may exert its effects by normalizing the dopaminergic function. Therefore, a possible mechanism may be related to an enhancement in the function of the remaining dopaminergic neurons. However, the possibility that the improvement in motor symptoms is due to changes to the pharmacokinetics of levodopa or other dopaminergic therapies cannot be excluded [53]. Nicotinamide riboside is also under investigation after the promising results of the NADPARK study, and four clinical trials are currently ongoing to confirm whether any clinical effect is evident.

## CONCLUSION

Our review highlights the potential benefits of using nutritional supplements and pharmacological compounds to target mitochondrial dysfunction in Parkinson’s disease patients. Despite some disappointing outcomes in RCTs, it is important to consider the potential benefits of exenatide and the promising results shown by nicotinamide riboside. Future research should focus on understanding the effects of nutritional supplements and pharmacological compounds in combination with existing drug therapies for PD, as well as optimising their delivery to maximise their symptomatic and neuroprotective effects. However, based on the current evidence, the clinical utility of compounds targeting mitochondrial dysfunctions in patients with PD remains under debate. Their use should be considered in a case-by-case scenario.

## Figures and Tables

**Table 1:** Summary of the literature review findings.

**Compound**	**Total Number of Papers Retrieved**	**Available RCTs**	**Ongoing Trials**
Coenzyme Q10	253	6	1 (NCT04152655)
Mitoquinone	10	1	0
N-Acetyl Cysteine	152	0	1 (NCT04459052)
Inosine	47	2	0
Nicotinamide riboside	140	1	4 (NCT05589766; NCT03568968; NCT05546567; NCT03808961)
Curcumin	149	1	0
Ursodeoxycholic acid	26	0	NCT03840005
Creatine	260	2	0
Exenatide	74	2	4 (NCT04232969; NCT04305002; NCT04269642; NCT04154072)
Pioglitazone	64	1	0
Isradipine	36	1	0
Methylene Blue	29	0	0
Dimethyl Fumarate	13	0	0
Triterpenoids	78	0	0
Riboflavin	15	0	0
Mito-Apocynin	8	0	0
SS31/SS20	0	0	0
Bezafibrate	0	0	0

**Note:** In the first column, a list of the identified compounds from the first literature search is provided. In the second column, the total number of papers retrieved discussing the neuroprotective or symptomatic effect of each compound in cellular models, animal models, or human participants with PD is reported. In the third and fourth columns, the number of published and ongoing RCTs in PD patients is given.

**Table 2:** Summary of RCTs.

**Study**	**Compound**	**Trial Design**	**Treatment Dose (Daily)**	**Disease Stage**	**No of Patients**		**Follow-up**	**Primary ** **Outcome Measure**	**Effect**
					**Active Treatment**	**Placebo**			
Shults *et al.*, 2002	Coenzyme Q10	Multicentre, parallel group	300 mg 600 mg 1200 mg	Early PD	64	16	16 months	Total UPDRS score	Less disability at 1200 mg daily (UPDRS score +6.69 *vs.* +11.99 in placebo)
Muller *et al.*, 2003	Coenzyme Q10	Monocentre, parallel group	360 mg	N.A	14	14	4 weeks	Total UPDRS score	No significant effect
Storch *et al.*, 2007	Coenzyme Q10	Multicentre, parallel group	300 mg	No motor fluctuations	44	47	3 months	UPDRS-II UPDRS-III	No significant effect
Parkinson study group QE3 investigators, 2014	Coenzyme Q10	Multicentre, parallel group	1200 mg 2400 mg	< 5 years disease duration	173	94	16 months	UPDRS-I UPDRS-II UPDRS-III	No significant effect
Yoritaka *et al.*, 2015	Coenzyme Q10	Multicentre	300 mg	Motor fluctuations	16	15	48 weeks	Total UPDRS score	Improvement (UPDRS score -4.2 *vs.* +2.9 in placebo)
			300 mg	Early PD	20	13	96 weeks		No significant effect
Li *et al.*, 2015	Coenzyme Q10 Creatine	Monocentre	300 mg 1000 mg	PD MCI	38	37	12 and 18 months	MOCA UPDRS-III	Delayed cognitive decline, MOCA (18.55 *vs.* 13.33 in placebo) No significant effect on UPDRS-III
Snow *et al*., 2010	Mitoquinone	Multicentre, parallel group	40 mg 80 mg	Drug naïve PD	89	41	12 months	Total UPDRS score	No significant effect
The Parkinson study group SURE-PD Investigators, 2014	Inosine	Multicentre, parallel group	Titrated to serum urate levels	Any PD (< 6 mg/dL of serum urate at baseline)	50	25	24 months	Safety, tolerability, and efficacy of urate elevation	Safe, tolerated, and effective in raising serum urate levels
The Parkinson study group SURE-PD3 Investigators, 2021	Inosine	Multicentre	Titrated to serum urate levels	Drug naïve PD (< 5.8 mg/dL of serum urate at baseline)	149	149	24 months	UPDRS-I UPDRS-II UPDRS-III	No significant effect (early termination)
Brakedal *et al.*, 2022	Nicotinamide riboside	Monocentre	1000 mg	Drug naïve PD	15	15	30 days	Cerebral penetration and metabolic response (^31^P-MRS, CSF, FDG-PET)	Increased brain nicotinamide riboside levels
Ghodsi *et al.*, 2022	Curcumin	Monocentre	80 mg	Any PD, on treatment	30	30	9 months	Total UPDRS score PDQ-39	No significant effect
Bender *et al.*, 2006	Creatine	Monocentre	20000 mg for 6 days 2000 mg for 6 months 4000 mg for the remainder	Any PD	40	20	24 months	DaTscan	No significant effect
NET-PD Investigators, 2015	Creatine	Multicentre, parallel group	10000 mg	All PD, on treatment	874	867	5 years	Clinical decline from baseline to 5 years follow up	No significant effect (early termination)
Aviles-Olmos *et al.*, 2013	Exenatide	Monocentre, parallel group	0.02 mg	Moderate PD	20	24 (no treatment)	14 months	UPDRS-III	Improvement (UPDRS score -2.7 *vs.* +2.2 in untreated group)
Athauda *et al.*, 2017	Exenatide	Monocentre, parallel group	2 mg (weekly)	Moderate PD, motor fluctuations	32	30	60 weeks	UPDRS-III	Improvement (UPDRS score -1 *vs.* +2.1 in placebo)
NET-PD, 2015	Pioglitazone	Multicentre, parallel group	15 mg 45 mg	Early PD	139	71	44 weeks	Total UPDRS score	No significant effect
STEADY-PD III, 2020	Isradipine	Multicentre, parallel group	10 mg	Early PD	169	165	36 months	UPDRS-I UPDRS-II UPDRS-III	No significant effect

**Abbreviations:** PD, Parkinson’s disease; MCI, mild cognitive impairment; N.A., not available; UPDRS, unified Parkinson's disease rating scale; PDQ-39, Parkinson's disease questionnaire; MOCA, Montreal cognitive assessment; 31P-MRS, 31-phosphorus magnetic resonance spectroscopy; CSF, cerebrospinal fluid; FDG-PET, fluorodeoxyglucose-positron emission tomography.

## LIST OF ABBREVIATIONS

NAD^+^: Nicotinamide Adenine Dinucleotide
PD: Parkinson’s Disease
UDCA: Ursodeoxycholic Acid
UPDRS: Unified Parkinson Disease Rating Scale
